# Potential Impact of Maternal and Newborn Health Improvements in Afghanistan: Projection of Mortality to 2030

**DOI:** 10.1007/s10995-025-04108-4

**Published:** 2025-05-13

**Authors:** Farzana Maruf, Hannah Tappis, Randolph Augustin, Thomas van den Akker, Yvonne Tam

**Affiliations:** 1 United States Agency for International Development, Washington, USA; 2 Athena Institute, Faculty of Science, Vrije Universiteit Amsterdam, Amsterdam, The Netherlands; 3 Center for Humanitarian Health, Johns Hopkins Bloomberg School of Public Health, Baltimore, MD USA; 4 Jhpiego, Baltimore, MD USA; 5 Department of Obstetrics and Gynecology, Leiden University Medical Centre, Leiden, The Netherlands; 6 Institute for International Programs, Johns Hopkins Bloomberg School of Public Health, Baltimore, MD USA

**Keywords:** Quality, Coverage, Mortality, Maternal and newborn health, Afghanistan

## Abstract

**Background:**

Despite remarkable progress, Afghanistan’s health sector continued to be hampered by chronic challenges undermining its performance including pervasive poverty and ongoing instability. At present, many pregnant women remain vulnerable because of low access to antenatal care, postnatal care, and skilled birth attendance.

**Objective:**

To illustrate the potential impact that continued improvements in maternal and neonatal health can have in terms of lives saved, and progress towards development goals. More nuanced modeling to consider the current quality of services is needed to inform resource mobilization and allocation decisions in a constrained fiscal space.

**Results:**

If coverage of evidence-based neonatal and maternal interventions reaches 90% of those in need by 2030, the neonatal mortality rate would drop from 36 to 16 per 1,000 live births, and the maternal mortality ratio from 638 to 237 per 100,000 live births. These reductions would mostly be driven by increases in coverage of interventions during childbirth.

**Conclusion:**

Tenacity, innovation, reinvigorated commitment, and continued financial resources are critically needed from the international health community and local government to avoid needless deaths and save lives.

**Supplementary Information:**

The online version contains supplementary material available at 10.1007/s10995-025-04108-4.

## Significance

This study presents more nuanced results that could be used for advocacy and prioritization of resources to inform decision-making amidst current fiscal and operational constraints. Meanwhile, it serves as a call upon the international health community and local government to maintain financial resources, tenacity, and innovation to avoid needless deaths and build resilience for women and children in Afghanistan. Previous studies modeling the impact of scaling up maternal and newborn health interventions in Afghanistan assumed all services are implemented with high quality. Fragile countries facing similar contextual challenges can use the tool and learnings as a means for advocacy, donor coordination, and prioritizing resources within constrained fiscal space to improve the quality and coverage of maternal and newborn health services and prevent mortality and morbidity.

**Supplementary Information:**

The online version contains supplementary material available at 10.1007/s10995-025-04108-4.

## Introduction

Achieving targets for the Sustainable Development Goals (SDGs) for 2030 will require not only rapid accelerated change but also robust linkages established across many sectors (Chou et al., 2019). Improving the survival chances of women and their newborns persists as an unfinished agenda facing the global community.

Despite pervasive poverty and ongoing instability, Afghanistan made remarkable gains in health, development, and access to essential maternal, newborn, and child health services from 2003 to 2020 (Ministry of Healthcare, 2020). Services at all public facilities in Afghanistan were standardized including a Basic Package of Health Services for primary healthcare facilities introduced in 2003 and an Essential Package of Hospital Services introduced in 2005. All public health facilities, from basic health centers to specialty hospitals, are expected to provide basic emergency obstetric and newborn care (Newbrander et al., 2014).

To reinforce political attention on maternal, newborn, and child health (MNCH), Afghanistan committed to implementing the Global Strategy for Women’s and Children’s Health in 2010 (*Secretary-General Calls for Broad Partnership to Support Global Strategy for Women’s*,* Children’s Health*,* 2023*). The country pledged—as part of *A Promise Renewed*—to act to accelerate progress on MNCH and held a national Call to Action event in 2015. Afghanistan signed on to the global Every Newborn Action Plan, launched a national Reproductive, Maternal, Newborn, Child, and Adolescent Health strategy in 2017, and adopted quality of care policies and approaches including the 2016 WHO standards for improving quality of maternal and neonatal care at health facilities that provide a series of clinical and experiential standards (Jhpiego, 2017; MoPH 2017; World Health Organization, 2014).

However, the country still has some of the highest maternal and neonatal mortality rates worldwide (WHO et al., 2023). Many pregnant women remain vulnerable because of low access to antenatal care, skilled birth attendants, and postnatal care; more than one-third of births still take place at home (KIT Royal Tropical Institute; Afghanistan National Statistics and Information Authority, 2019). An estimated 20% of deliveries require lifesaving emergency interventions, yet these are not readily accessible to most women and their newborns - inequities, gender norms, and financial barriers exist, both in rural and urban settings. Assessments conducted before 2021 documented provision of substandard care in public and private sectors. The experience of care reported by clients often accompanies the substandard provision of care and is rarely measured in routine health systems (Jhpiego, 2017).

Due to regime change on August 15, 2021, Afghanistan is confronting an unprecedented humanitarian and economic crisis with a risk of systemic collapse and humanitarian catastrophe that threatens many of the development gains of the last 20 years (*United Nations Office for the Coordination of Humanitarian Affairs and WHO*, 2022). Over 24.4 million Afghans out of 32 million need humanitarian assistance, of whom 18.1 million require immediate health care (UNFPA, 2023).

Sustaining gains in the health sector improvements over the last decade, and addressing gaps in quality of care, will require substantial resource investments. However, Afghanistan faces significant fiscal constraints as donor financing has declined and domestic revenue mobilization remains very limited. There is a high out-of-pocket expenditure rate (77%) in the health sector, and a significant portion is spent on drugs and medical products (Ministry of Public Health Afghanistan, 2021). Underfunding of health systems, cutbacks in aid, shortfalls in resources, and lack of competent healthcare providers are undermining efforts to increase resiliency and reduce equity gaps in many settings, including in Afghanistan (Jhpiego, 2017; UNFPA, 2022; Zarocostas, 2023).

Mathematical modeling is often used to analyze the impacts of potential investments on health outcomes. Previous studies in Afghanistan have modelled the impact of scaling up coverage of maternal and newborn health interventions on lives saved, assuming all services are implemented with high quality (*UNICEF. Investing in Newborn in South Asia*, 2021). More nuanced modeling that takes into account the current quality of services is needed to inform resource mobilization and allocation decisions in such a constrained fiscal space.

## Methods

### Lives Saved Tool

The Lives Saved Tool (LiST) is a multi-cause mortality model for maternal, neonatal, child, and stillbirths. It models the impact of scaling up coverage of proven health interventions that can be feasibly implemented in low- and middle-income countries (LMICs) to reduce cause-specific mortality directly, or via reducing risk factors for cause-specific mortality. These causal pathways can be visualized interactively at https://listvisualizer.org/. Any changes in distal factors due to policies and systems, changes in intermediate factors due to household and individual context, or programs and services, are accounted for in the changes in coverage of health interventions. Detailed information on the data sources, methods, and assumptions used in LiST has been published elsewhere (Walker et al., 2013). The model is publicly available at www.livessavedtool.org, and additional information on resources to use the model or research that used the model is curated on the website. Version 6.19 was used for this analysis.

The main inputs needed for modeling are a baseline description of the health status of Afghanistan, including population size and structure, mortality rates, and causes of death. The changes in coverage and the effectiveness of the interventions, applied to the baseline descriptions of the country, are used to compute the cause-specific reduction in mortality. The main modeling inputs and their sources are listed in Supplemental Table 1. The main outputs of the model are the number of deaths, additional lives saved, and mortality rates. Supplemental Figs. 1 and 2 show interventions that reduce maternal and neonatal mortality.

Neonatal and maternal mortality reduction is primarily driven by interventions delivered during antenatal care, intrapartum care at health facilities, and postnatal care, where their utilization or service contact rates are available from household surveys. However, as the reduction of cause-specific mortality is linked to the effectiveness of interventions delivered during these contacts, we need to estimate the true coverage of these interventions. This is estimated by multiplying the proportion of pregnant women who utilized antenatal care or gave birth at facilities (reported in nationally representative household surveys) by the proportion of facilities ready to deliver the interventions (reported in nationally representative facility surveys). At the time of analysis, Afghanistan lacks household and facility surveys that can be temporally linked to compute a readiness estimate for interventions delivered during ANC, childbirth, and postnatal care. Instead, we computed median readiness estimates from 17 countries with linked household and facility surveys. This readiness estimate was then multiplied by the Afghanistan service utilization trend to produce a readiness-adjusted coverage trend of the intervention. In addition, we were able to compute an annual rate of change for readiness estimates, using trend coverage of Afghanistan’s antenatal care contents, including blood pressure, blood samples, and urine samples taken from household surveys. We applied this annual rate of change to the readiness estimates to create a readiness estimate trend for antenatal care and childbirth interventions. Methods used to estimate quality-adjusted coverage (readiness-adjusted coverage) have been previously published), and more details on estimating trends of quality can be found in this technical note. We also reviewed various publications based on analyses of the 2016 Afghanistan National Maternal and Newborn Health Quality of Care Assessment and the 2019 Afghanistan Service Provision Assessment (MoPH, 2019) to ensure our readiness estimates used for modeling are comparable to those in recent publications and reports (Ansari et al., 2020; Atiqzai et al., 2019; Lydon et al., 2021).

### Coverage Scenarios

We designed scenarios to quantify the impact of improving quality and improving quality and utilization to reach high coverage.

Quality is defined as the readiness of facilities to deliver interventions, and utilization is defined as the proportion of pregnant women that attended ANC or delivered in facilities. Quality and utilization are the two components that drive coverage increase. Coverage is defined as the proportion of women or children that received the interventions among those that need it.

#### Scenario 1

What is the impact if one focuses on improving the quality of the interventions by making most of the facilities ready to deliver those interventions? This question can be answered with results from a scenario where 90% of the facilities delivering care during pregnancy and childbirth are ready to deliver the interventions, with no change in utilization of ANC or facility birth rates.

#### Scenario 2

What is the impact on maternal and neonatal health if interventions were to reach most that needed them? This question can be answered with results from a scenario where 95% of the facilities delivering care during pregnancy and childbirth are ready to deliver the interventions, and when 95% of the pregnant women attended ANC and delivered in facilities, resulting in 90% (95% x 95%) coverage of the interventions due to high quality of interventions and high level of utilization of facilities that deliver the interventions.

Inputs used for each of these scenarios can be found in Supplemental Table 1, and baseline quality, utilization, and coverage estimate by interventions can be found in Supplemental Table 2.

## Results

### Scenario 1: Impact of Quality of Interventions Reaching 90%

**Fig. 1:**
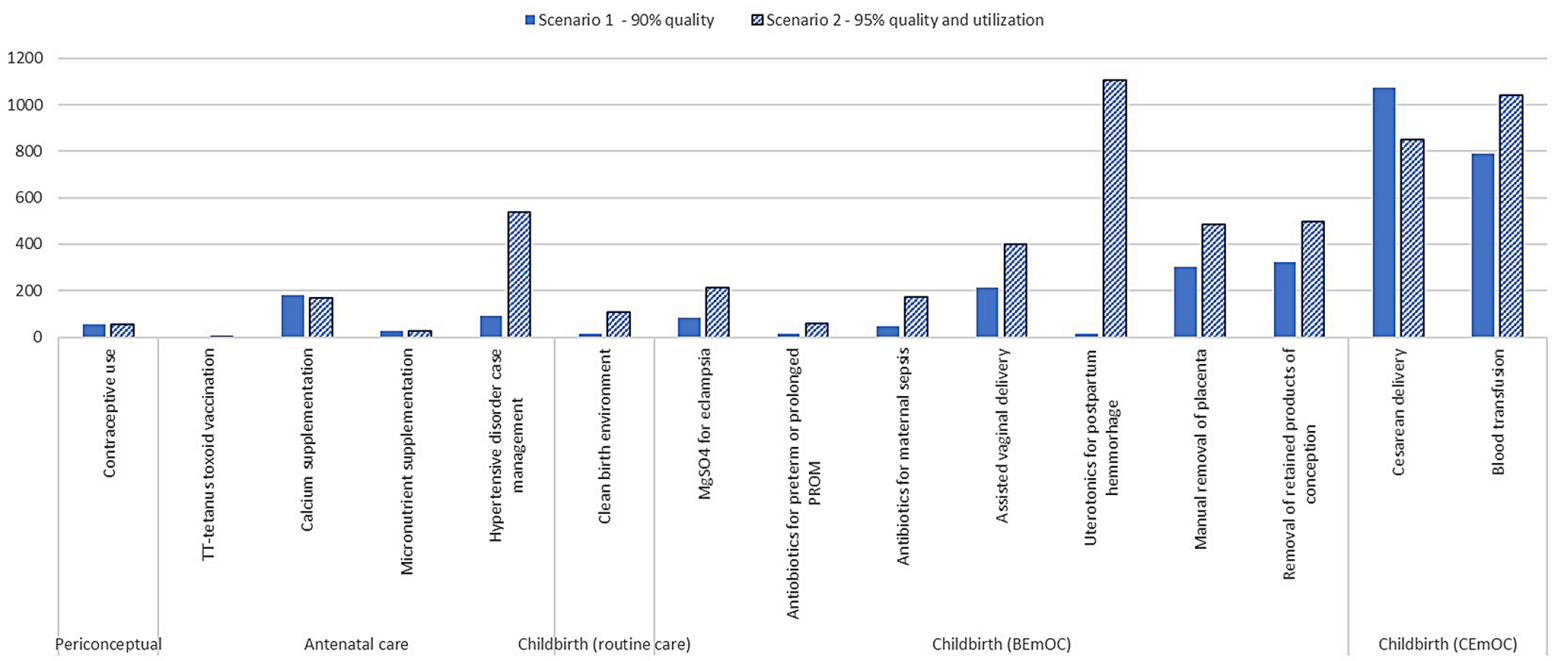
Total maternal lives saved in 2030 in Afghanistan if the quality of neonatal and maternal interventions reaches 90% by 2030 (scenario 1) and if the coverage of neonatal and maternal interventions reaches 90% by 2030 (scenario 2)

When intervention quality reaches 90%, we project 5,228 yearly maternal lives saved, compared to no change in quality (Fig. 1). The interventions that led to the number of maternal lives saved are Cesarean delivery, followed by blood transfusion and removal of retained products of conception. In terms of grouping interventions by the timing they are delivered, 58% of maternal lives saved are attributed to comprehensive emergency obstetric care (CEmOC) specific interventions during childbirth and all interventions during childbirth account for 89% of the total maternal lives saved.

**Fig. 2:**
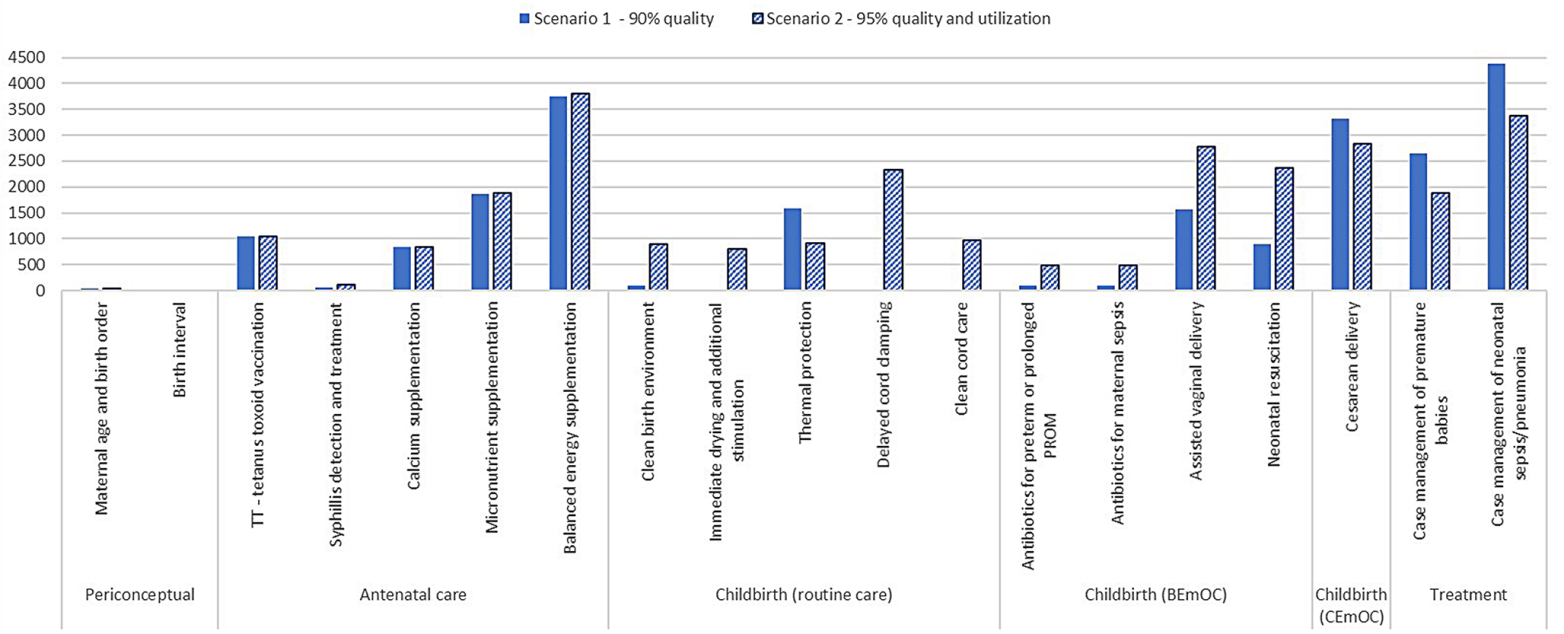
Total neonatal lives saved in 2030 in Afghanistan if quality of neonatal and maternal interventions reaches 90% by 2030 (scenario 1) and if the coverage of neonatal and maternal interventions reaches 90% by 2030 (scenario 2)

When the quality of interventions reaches 90% by 2030, we project 22,275 yearly additional neonatal lives saved, compared to no change in quality (Fig. 2). The interventions that led to the number of neonatal lives saved are case management of neonatal sepsis/pneumonia, followed by balanced energy supplementation and Cesarean delivery. In terms of grouping interventions by the timing they are delivered, 34% of neonatal lives saved are attributed to antenatal care interventions. Interventions during childbirth including interventions for routine care, basic emergency obstetric care (BemOC), and CEmOC account for 34% of the total neonatal lives saved. Of note, the estimated current quality of interventions such as clean cord care, thermal protection, immediate drying, and additional stimulation are at or higher than 90% and were kept as is over time.

### Scenario 2: Impact of Quality and Utilization Reaching 95%, Resulting in 90% Coverage of Interventions

When interventions were received by 90% of those that needed them by 2030 due to high levels of quality and utilization, we project 5,716 yearly additional maternal lives saved, compared to no change (Fig. 1). The interventions that contributed to the greatest number of maternal lives saved are uterotonics for postpartum hemorrhage treatment followed by blood transfusion and Cesarean delivery. In terms of grouping interventions by the timing they are delivered, 51% of maternal lives saved are attributed to BEmOC-specific interventions during childbirth and all interventions during childbirth account for 86% of the total maternal lives saved.

When effective interventions reach the most that needed them by 2030, we project 27,890 yearly additional neonatal lives saved, compared to no change in coverage (Fig. 2). The interventions that contributed to the greatest number of neonatal lives saved are balanced energy supplementation, followed by case management of neonatal sepsis/pneumonia, and Cesarean Section. In terms of grouping interventions by the timing, they are delivered, 28% of neonatal lives saved are attributed to antenatal care interventions. Interventions during childbirth including interventions for routine care, BEmOC, and CEmOC account for 53% of the total neonatal lives saved.

**Fig. 3:**
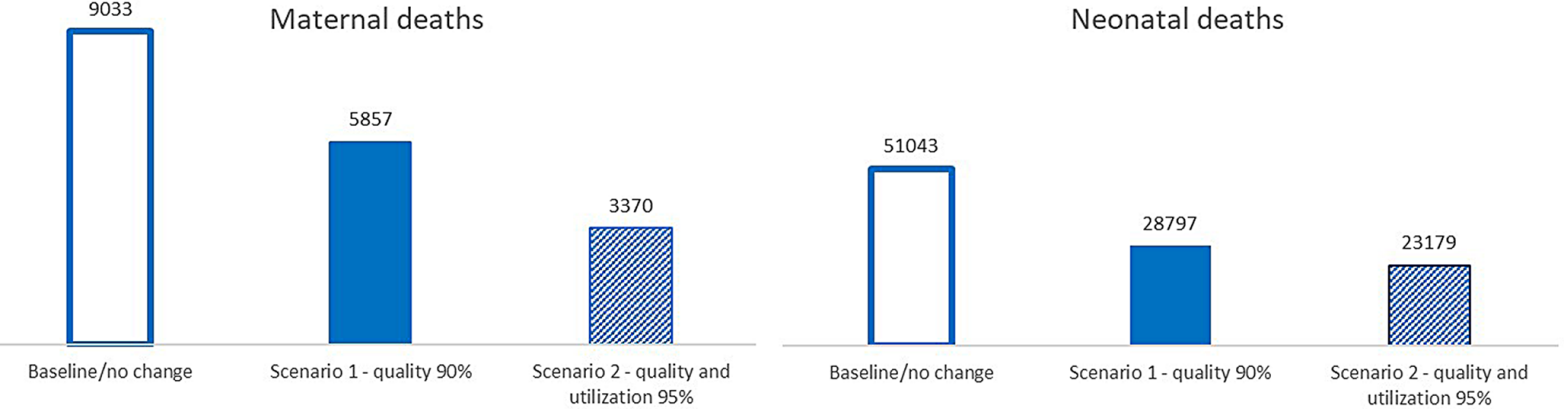
Projected count of maternal and neonatal deaths in 2030 if the quality of neonatal and maternal interventions reaches 90% by 2030 (scenario 1) and if the coverage of neonatal and maternal interventions reaches 90% by 2030 (scenario 2)

If coverage of all proven neonatal and maternal interventions reaches 90% of those that needed them by 2030, LiST projects the maternal mortality ratio (MMR) reduces from 638 to 237 maternal deaths per 100,000 live births, and the neonatal mortality rate to reduce from 36 to 16 neonatal deaths per 1,000 live births (Fig. 3). These improvements in coverage of interventions bring the country closer to the SDG goals of 12 or fewer neonatal deaths per 1,000 live births and less than 70 maternal deaths per 100,000 live births by 2030.

## Discussion

Our modelling shows there are high positive returns on investment in all proven interventions to achieve the targets for maternal and newborn mortality reduction in Afghanistan. Increasing both the quality and utilization of health interventions at health facilities are key factors to achieve high coverage and therefore impact of interventions.

Evidence suggests if those seeking medical attention arrived at facilities reimagined with adequate resources and receive high-quality care including timely primary health care referrals, almost one-quarter of the maternal and neonatal deaths and stillbirths would be preventable (Chou et al., 2019). Greater investments are needed to address missed opportunities, and enhance quality respectful care practices across health cadres, intrinsically linked to the uptake of antenatal care, skilled birth attendance, postnatal care, and continuity of maternal and newborn care in Afghanistan.

These findings are in line with previous studies on mortality associated with cesarean section (CS) which could be partly due to women coming late for obstetric care in Afghanistan. Increasing availability and utilization of CS requires focus on quality, such as encouraging use of partographs and improving decision-making and documentation (Kim et al., 2012). Afghanistan has a low CS rate of 6% nationwide that needs due attention (KIT Royal Tropical Institute; Afghanistan National Statistics and Information Authority, 2019). It is important to perform CS for those women who are in need, otherwise, unnecessary surgery will increase the risk of death among women (Betran et al., 2021).

In the absence of critical and comprehensive investments, maternal mortality will rise in Afghanistan. According to one estimate, the MMR might increase by 50% from 638 to 963 deaths per 100,000 live births in 2025 (*United Nation Population Funds Afghanistan*, 2022). Access to basic and essential lifesaving interventions before, during, and after childbirth by a skilled health professional and breastfeeding could prevent nearly all these deaths (USAID, 2023). This is critical information to guide investment decisions as governments and donors prioritize where and how to spend their limited resources, further constrained in the current context of the COVID-19 pandemic, conservative economic assumptions, and fragility. To strengthen value for money, it is paramount to examine and increase investments in preventive health services (Ministry of Public Health Afghanistan, 2021). Afghanistan’s health financing system is highly dependent on donor funds. If the current levels of financing for the health sector cannot be increased, these must at least be maintained. Since no significant progress has been made over the last two decades to shift from out-of-pocket spending to a mechanism of prepayment or taxation to increase public funding, it is highly unlikely that more sustainable funding mechanisms will be addressed in the current context. Considering Afghanistan’s limited capacity to generate additional domestic revenue, it is imperative that external financing stays at current level or be expanded to save the lives of vulnerable women and children. Increasing the national budget allocation to the health sector is also necessary to maintain life-saving primary and essential health service delivery, particularly in rural areas (Basij-Rasikh et al., 2024; Royal Tropical Institute, World Bank, 2023). Achieving the SDG targets requires a much greater acceleration in the annual rates of reduction for maternal and child mortality than the path they are currently on. It would require consistent, long-term, and high-level political and financial commitments and resource allocations, both by local government and donors (Barış et al., 2021).

Another question is whether it is realistic for Afghanistan to achieve 90% coverage of interventions during childbirth, or nutritional interventions during the antenatal period. Given the significant political shift in Afghanistan following the Taliban’s takeover in August 2021, assessing the level of support (or lack of opposition) from the current leadership for proposed interventions is crucial. International assistance, including humanitarian aid, healthcare, and infrastructure support continues in public and private sectors (United Nations Office for the Coordination of Humanitarian Affairs, 2024). Health service delivery rates fell by approximately 15% after the Taliban take over in 2021. The reinstatement of donor financing has stabilized access to health care since early 2022 and maintained the delivery of essential health countrywide, including for women and girls as female health workers, particularly female doctors, midwives, and nurses have continued working without any interruptions. Despite significant challenges, Afghanistan’s health facilities provide valued services to their communities and women are allowed to seek care for reproductive, maternal, and childbirth care. The staffing level remains consistent in all facilities with high retention rates; however, shortages exist for specific specialist positions such as general practitioners and female pediatric specialists. Nearly all the required equipment was available, but shortages of certain medicines remain a concern that needs to be addressed. (*Afghanistan Multiple Indicator Cluster Survey (MICS)*,* 2022–2023*; Andersen et al., 2023). Current evidence doesn’t suggest a significant variation in coverage of maternal and child health services compared to the Pre-Taliban takeover, in particular for antenatal care and skilled birth attendance (*Afghanistan Multiple Indicator Cluster Survey (MICS)*,* 2022–2023*).

Previous studies in Afghanistan flagged the importance of supportive supervision systems, in-service training, continuous mentoring, and job rotation, which could help health providers possess the knowledge, skills, and attitudes required to provide quality emergency obstetric and newborn care services A comprehensive, trained, protected, well-equipped, and supported human resource is foundational to expanding equitable access to health services and health care across the country (Ansari et al., 2019; Deussom et al., 2022; Maruf et al., 2021a, b).

Studies highlight the critical role of social accountability in changing the power dynamics between the health systems and patients and their families. For instance, it can increase provision of respectful care and improved health outcomes and trust that lead to more use of the healthcare system, minimized medication adherence issues, and even improved working conditions of health workers by reducing burnout (Streifel, 2022). More investments are required to strengthen social accountability e.g. score cards and citizen charters in Afghanistan to improve the health and well-being of women and children.

Past studies in Afghanistan recommended improvement of maternal and newborn health record keeping, health management information systems including birth and civil registration, and vital statistics that require robust political support and investment at various levels. This will support real-time clinical and political decision-making, priority setting, and accountability (Maruf et al., 2021a, b).

It is estimated that more than 50% of Afghanistan’s disease burden is influenced by factors that lie beyond the scope of health care, that is, by the social determinants of health and environmental conditions, such as food security, education, employment and income, safety nets, and humanitarian actors (Royal Tropical Institute; World Bank, 2023). Engaging with the private sector is crucial to addressing many key drivers of maternal and child mortality in Afghanistan. The sector has the potential to become a sustainable source of economic growth and stability in the face of increasing political uncertainty. This partnership plays an important role in the provision of essential medicines, medical supply chain, and as a provider of quality healthcare services and needs further attention to leverage and regulate the sector (USAID, 2019; World Health Organization, 2022).

There are various cross-cutting interventions to address key determinants of health and nutrition by improving coordination between both development and humanitarian partners to make health systems more resilient and responsive. While the health sector is central to many of these interventions, it cannot achieve the required goals without the active contribution of and collaboration with other sectors such as water, sanitation and hygiene, nutrition, gender, education. Building political support for health and nutrition improvements requires strategic navigation of local power structures and trust-building within communities. Engaging influential tribal elders, religious leaders, scholars, and community representatives is crucial to securing advocacy for maternal and newborn health initiatives. Programs should align with cultural norms and Afghan traditions to ensure acceptance. Additionally, partnering with the private sector, NGOs, professional associations, civil society organizations, and local actors is essential to effectively address maternal and newborn health needs and enhance local ownership of interventions. (Lugten et al., 2023; Ministry of Public Health 2020; Vision for Health System Strengthening, 2030, *USAID*).

Thus, tenacity, innovation, reinvigorated commitment, and continued financial resources are critically needed from the international health community and local government to avoid needless deaths and build resilience through sustained health outcomes for women, children, and families.

A major limitation of this analysis is the lack of accurate baseline inputs needed for modeling. We explored modeling potential impacts on mortality if there were declines in coverage and quality of services after the change in government in August 2021 but did not have data to do so. Results from ongoing surveys when available may strengthen our analyses. Besides that, our target coverage was aspirational, and the results of this analysis were meant for advocacy purposes, justifying through the lens of meeting most of the needs of neonates and women. As more current data on health service quality and utilization become available, modeling potential impacts of a broader range of investment scenarios could help guide allocation of scarce resources and strengthen advocacy for continued attention to Afghan women and children health.

## Electronic supplementary material

Below is the link to the electronic supplementary material.

Supplementary Material 1

## Declarations

### Disclaimers

The views and opinions expressed in this article are those of the authors and not necessarily the views and opinions of the United States Agency for International Development.
