# Supplementary material for: Potential Impact of Maternal and Newborn Health Improvements in Afghanistan: Projection of Mortality to 2030
Source: Matern Child Health J. 2025 May 13;29(6):791–8. doi: 10.1007/s10995-025-04108-4 (PMC12206177; doi:10.1007/s10995-025-04108-4)

Table 1. List of main baseline inputs for modeling and their sources.

| Main baseline inputs for modeling | Source |
| --- | --- |
| Population | 2019 Revision of World Population Prospects, prepared by the Population Division of the Dept of Economic and Social Affairs of the UN Secretariat |
| Maternal mortality ratio | Estimates by WHO, UNICEF, UNFPA, World Bank Group, and the UN Population Division, published Sept 2019 |
| Neonatal mortality rate | UN Inter-agency Group for Child Mortality Estimation (IGME), published Oct 2020 |
| Causes of maternal death | Say L, Chou D, Gemmill A, et al. Global causes of maternal death: A WHO systematic analysis. Lancet Global Health 2014; 2(6): e323-33. |
| Causes of neonatal death | Liu L, Johnson HL, Cousens S, et al, for the Child Health Epidemiology Reference Group of WHO and UNICEF. Global, regional, and national causes of child mortality: an updated systematic analysis for 2010 with time trends since 2000. Lancet 2012; published online May 11. DOI:10.1016/S0140-6736(12)60560-1. |
| Coverage of interventions for most maternal and neonatal interventions | Demographic Health Survey 2015, Afghanistan Health Survey 2018 |
| Readiness-adjusted coverage of interventions delivered during antenatal care (ANC), delivery at health facilities, and postnatal care | Used median of readiness scores of 17 countries with linked household and facility surveys |

Table 2. Baseline level (%) of quality, utilization, and coverage per interventions

|  | **Baseline levels (%)** | | |
| --- | --- | --- | --- |
| **Interventions during pregnancy** | **Quality** | **Utilization** | **Coverage** |
| Antenatal care (at least 1 visit) |  | 63.8 |  |
| Antenatal care (at least 4 visits) |  | 20.9 |  |
|  |  |  |  |
| **Routine** |  |  |  |
| TT - Tetanus toxoid vaccination |  |  | 39.6 |
| Syphilis detection and treatment | 24.7 | 63.8 | 15.8 |
| **Nutritional** |  |  |  |
| Calcium supplementation |  |  | 0.0 |
| Micronutrient supplementation (iron and multiple micronutrients) |  |  | 46.8 |
| Balanced energy supplementation |  |  | 0.0 |
| **Case management** |  |  |  |
| Hypertensive disorder case management | 24.0 | 20.9 | 5.0 |
| Diabetes case management | 18.7 | 20.9 | 3.9 |
|  |  |  |  |
|  | **Baseline levels (%)** | | |
| **Interventions during childbirth** | **Quality** | **Utilization** | **Coverage** |
| Health facility delivery |  | 56.3 |  |
|  |  |  |  |
| **Routine care** |  |  |  |
| Clean birth environment | 82.0 | 56.3 | 46.2 |
| Immediate drying and additional stimulation | 91.6 | 56.3 | 51.5 |
| Thermal protection | 98.8 | 56.3 | 55.6 |
| Delayed cord clamping | 0.0 | 56.3 | 0 |
| Clean cord care | 95.4 | 56.3 | 53.7 |
| **Basic emergency care** |  |  |  |
| MgSO4 for eclampsia | 71.5 | 56.3 | 40.3 |
| Antibiotics for preterm or prolonged PROM | 74.8 | 56.3 | 42.1 |
| Antibiotics for maternal sepsis | 74.8 | 56.3 | 42.1 |
| Assisted vaginal delivery | 25.3 | 56.3 | 14.2 |
| Neonatal resuscitation | 55.0 | 56.3 | 31.0 |
| Uterotonics for postpartum hemorrhage | 89.4 | 56.3 | 50.3 |
| Manual removal of placenta | 37.4 | 56.3 | 21.1 |
| Removal of retained products of conception | 33.1 | 56.3 | 18.7 |
| Induction of labor for pregnancies lasting 41+ weeks | 1.8 | 56.3 | 1.0 |
| **Comprehensive emergency care** |  |  |  |
| Cesarean delivery |  |  | 25.6 |
| Blood transfusion | 12.6 | 56.3 | 7.1 |
|  |  |  |  |

Figure 1. Proven neonatal interventions and their impact on cause-specific mortality in LiST. Visit <https://listvisualizer.org/> for details.
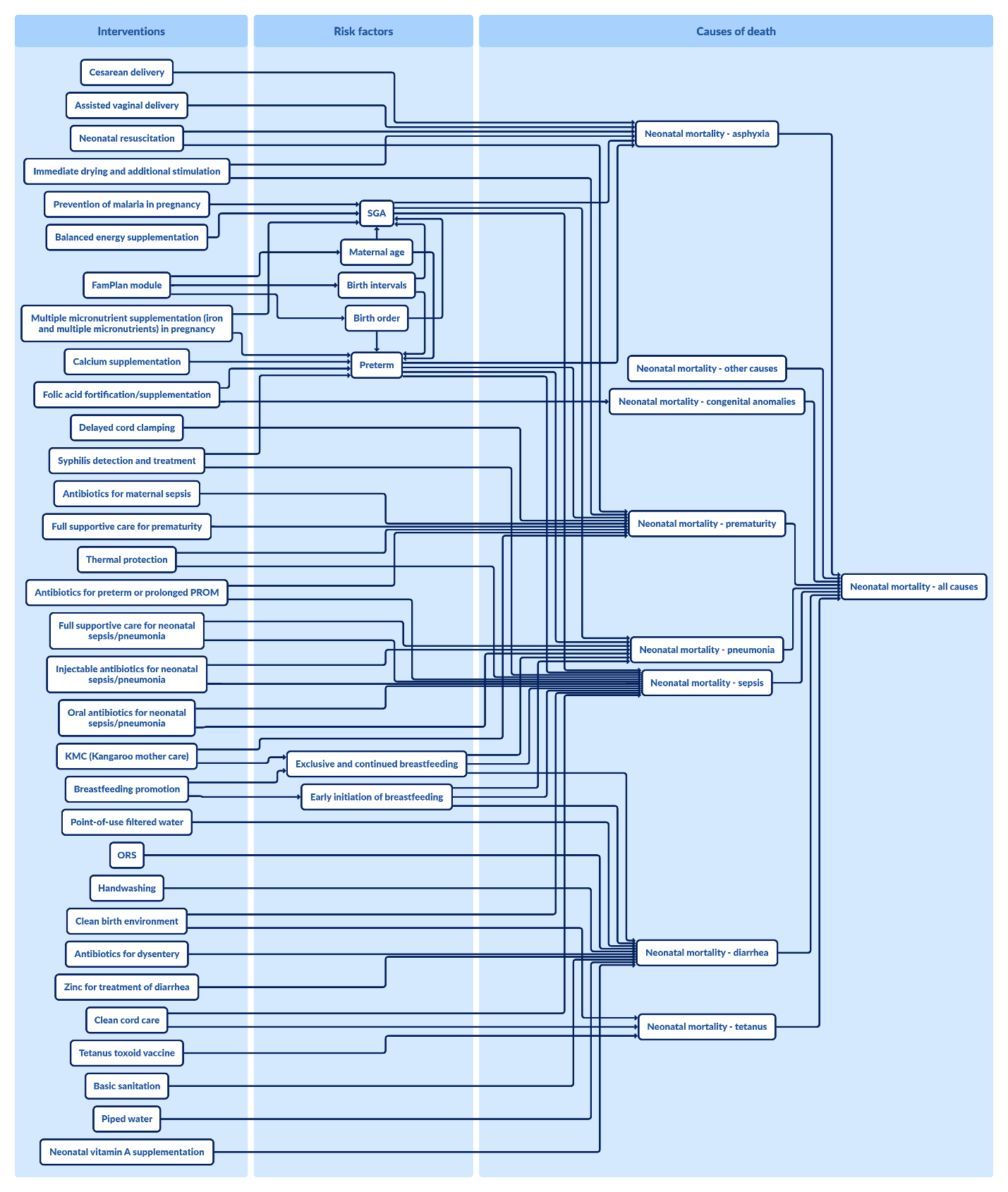


Figure 2. Proven maternal interventions and their impact on cause-specific mortality in LiST. Visit [https://listvisualizer.org/](https://listvisualizer.org/%20) for details.


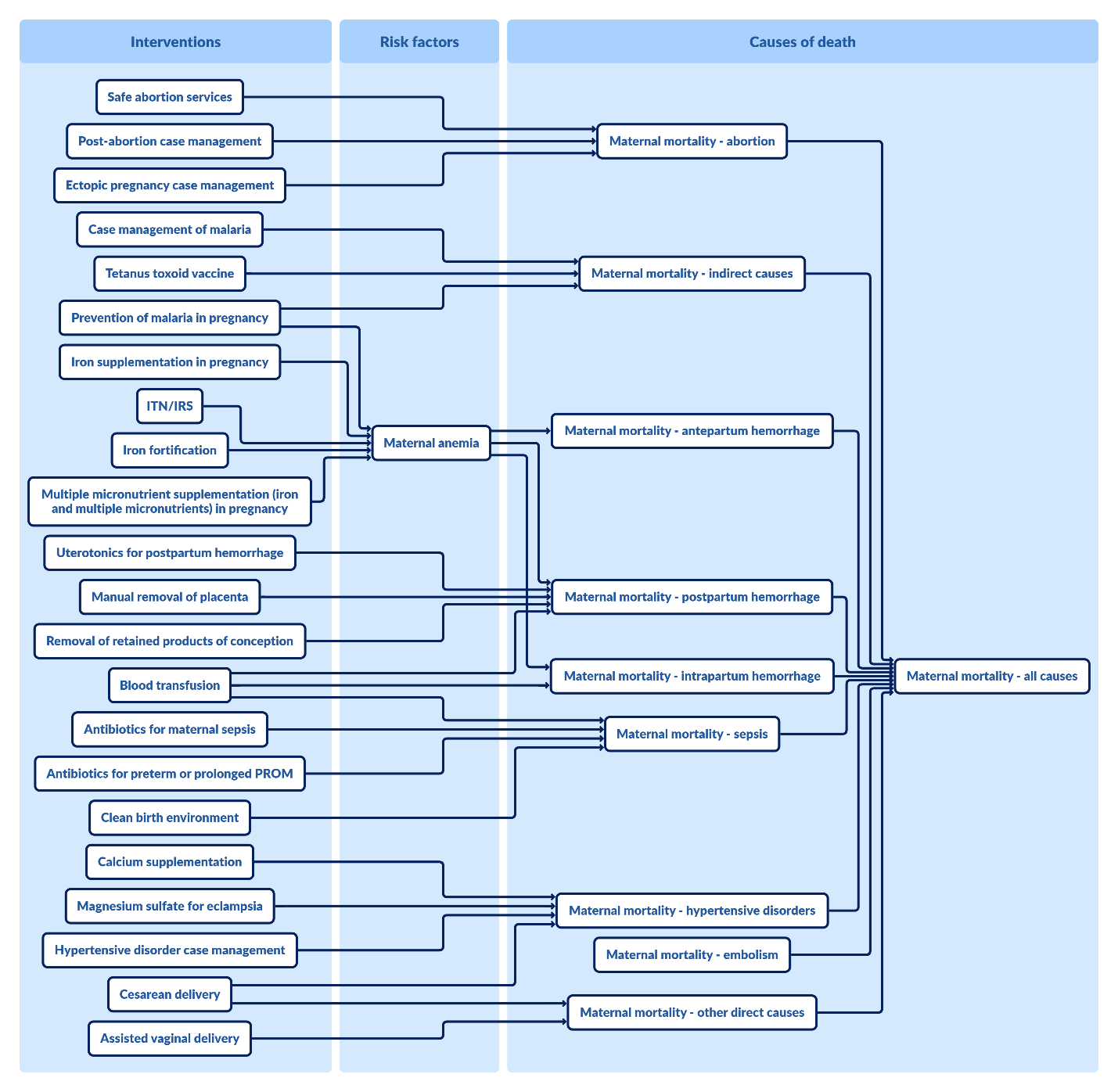

Supplement: Supplementary file 1 — Supplementary Material 1 [file 10995_2025_4108_MOESM1_ESM.docx]
